# Strengths and Limitations of 16S rRNA Gene Amplicon Sequencing in Revealing Temporal Microbial Community Dynamics

**DOI:** 10.1371/journal.pone.0093827

**Published:** 2014-04-08

**Authors:** Rachel Poretsky, Luis M. Rodriguez-R, Chengwei Luo, Despina Tsementzi, Konstantinos T. Konstantinidis

**Affiliations:** 1 School of Civil and Environmental Engineering, Georgia Institute of Technology, Atlanta, Georgia, United States of America; 2 School of Biology, Georgia Institute of Technology, Atlanta, Georgia, United States of America; 3 Center for Bioinformatics Computational Genomics, Georgia Institute of Technology, Atlanta, Georgia, United States of America; Universidad Miguel Hernandez, Spain

## Abstract

This study explored the short-term planktonic microbial community structure and resilience in Lake Lanier (GA, USA) while simultaneously evaluating the technical aspects of identifying taxa via 16S rRNA gene amplicon and metagenomic sequence data. 16S rRNA gene amplicons generated from four temporally discrete samples were sequenced with 454 GS-FLX-Ti yielding ∼40,000 rRNA gene sequences from each sample and representing ∼300 observed OTUs. Replicates obtained from the same biological sample clustered together but several biases were observed, linked to either the PCR or sequencing-preparation steps. In comparisons with companion whole-community shotgun metagenome datasets, the estimated number of OTUs at each timepoint was concordant, but 1.5 times and ∼10 times as many phyla and genera, respectively, were identified in the metagenomes. Our analyses showed that the 16S rRNA gene captures broad shifts in community diversity over time, but with limited resolution and lower sensitivity compared to metagenomic data. We also identified OTUs that showed marked shifts in abundance over four close timepoints separated by perturbations and tracked these taxa in the metagenome vs. 16S rRNA amplicon data. A strong summer storm had less of an effect on community composition than did seasonal mixing, which revealed a distinct succession of organisms. This study provides insights into freshwater microbial communities and advances the approaches for assessing community diversity and dynamics *in situ*.

## Introduction

A key step in understanding microbial community structure, dynamics, and how organisms might influence or be influenced by their surroundings is to classify DNA sequences taxonomically or phylogenetically. To date, most studies of microbial communities in systems ranging from the open ocean to soil to the human gut have depended on a single gene, the 16S small subunit ribosomal RNA (rRNA) gene [1]–[4]. Massively parallel sequencing methods are increasingly being applied to the characterization of microbial communities based on amplification of this gene and have led to a better appreciation of extant biodiversity [5]; however, the 16S rRNA -based techniques are known to be limited by the short read lengths obtained, sequencing errors [6], [7], differences arising from the different regions chosen [8], and difficulties in assessing operational taxonomic units (OTUs) [9]. Furthermore, the use of a single marker gene to assess diversity is challenging, given the prevalence of horizontal gene transfer and the difficulty inherent in defining bacterial species [10]–[12] as well as the limited resolution of the 16S rRNA gene among closely related species. Recently, 16S rRNA gene amplicon sequencing was compared to metagenomic data from synthetic communities [13], but to our knowledge, there has been no systematic evaluation of high-throughput 16S rRNA gene sequencing involving multiple sequencing and PCR replicates from natural microbial communities. Here, we coupled detailed analyses of replicate 16S rRNA gene datasets to comparisons with companion community shotgun metagenomics data from the same samples.

Metagenome approaches are commonly used to describe microbial communities in different systems, e.g., [14]–[16], without the biases inherent to PCR amplification of a single gene, although it remains a challenge to accurately infer taxonomic origin from metagenomic reads [17]. Whole genome shotgun (WGS) metagenomic approaches provide robust estimates of microbial community composition and diversity without the need to target and amplify a specific gene. However, differences in sequencing platforms, DNA preparation methods, and the complexity of the samples being studied can possibly lead to different or biased observations [6], [18], [19]. Furthermore, phylogenetic classification of microbes using WGS is seldom coupled to 16S-based classification, and a few recent studies doing so have identified discrepancies between the different classification methods, usually with regard to the level of resolution obtained [20], [21].

Here, we focused on evaluating the bacterioplankton composition and short-term variability in an important, temperate freshwater lake in the Southeast USA, Lake Lanier. Lake Lanier is the source of drinking water for metropolitan Atlanta and is a popular recreational area, especially during the summer months. Freshwater microbial communities have been shown to change over time in many different systems [22]–[25], influenced by a variety of environmental factors such as pH, temperature, and water retention time [26]–[28], but little is known about microbial temporal dynamics or responses to natural perturbations such as strong storm events in southern temperate lakes such as Lake Lanier. Understanding the microbial community composition, variation, and metabolic potential of Lake Lanier will help discern the sensitivity and responsiveness of this community to potential perturbations as well as address the gap of knowledge of freshwater lake communities. We examined the microbial community with 16S rRNA gene and metagenomic sequencing while assessing the reproducibility and potential biases of the 16S-based approach by comparing multiple PCR (i.e., same template DNA with independent 16S rDNA amplifications) and sequencing (i.e., same DNA sequenced independently) replicate datasets from four different timepoints separated by two different potential perturbations: a summer storm event and the beginning of the fall turnover. We further compared this 16S-based information to that from functional genes and 16S rRNA gene fragments recovered in companion metagenomic datasets to determine the extent to which an amplicon approach influences our ecological inferences and to evaluate the strengths and limitations of these common community characterization approaches. In addition to providing information about the technical variability of the 16S rRNA gene amplicon approach, we also gained new insights into the microbial ecology of the system, specifically about how certain physiochemical changes might influence bacterioplankton communities as well as how community composition changes over time. This work is also part of a larger, long-term effort to characterize the microbial community of Lake Lanier [29].

## Methods

### Sample Description

Lake Lanier is a seasonally stratified lake situated about 80 km northeast of Atlanta, GA at the headwaters of the Apalachicola-Chattahoochee-Flint River basin. When full, the reservoir covers nearly 156 km^2^ and holds approximately 2.4×10^9^ m^3^ of water. It is used for drinking water, hydroelectric power generation, flood control, run-off management, and recreation. Samples were collected from below the Browns Bridge at Lake Lanier (34°N 15′ 43″, 83°W 57′ 7″) at four time points in 2009: three centered around an August 27 storm event (August 26, AUG1; August 28, AUG2; and September 7, SEPT) and one during the fall mixing event in November (November 8, NOV). No specific permissions were required for this sampling location, nor did our field study involve endangered or protected species. At each sampling point, a Water Quality Meter (Horiba) was used to measure water temperature, pH, conductivity, turbidity, dissolved oxygen, and total dissolved solids (Table S1 in File S1, Fig. S1 in File S1). A horizontal sampler (Wildco Instruments) was used to collect samples of planktonic microbial communities at 5 m, within the epilimnion, which is fairly uniform in temperature and fully oxygenated during summer stratification. Metagenomes from these same samples were analyzed previously [29].

### DNA Extraction

A total of 10 L of water was pre-filtered through ∼1.6 mm GF/A filters (Whatman) and cells were collected on 0.22 μm Sterivex filters (Millipore) using a peristaltic pump. Sterivex filters were stored at −80°C until DNA extraction. DNA was extracted as described in [29]. Briefly, filters were treated with lysis buffer (50 mM Tris-HCl, 40 mM EDTA, and 0.75 M sucrose) and incubated with 1 mg/ml lysozyme at 37°C for 30 min. Samples were subsequently incubated with 1% SDS, 10 mg/ml proteinase K, and 150 mg/ml RNAse for 2 h rotating at 55°C. DNA was extracted from the lysate with phenol and chloroform, precipitated with ethanol and eluted in TE buffer. DNA yield was about 1.5 μg per liter of water filtered. For the metagenome, ∼5 μg of the total DNA aliquot was sequenced using the Illumina GA-II sequencers at the Emory University Genomics Facility, providing paired-end reads with an average length of 100 bp (Table S4 in File S1). 16S rRNA gene amplicon pyrosequencing (see below) was run on the GS-FLX 454 Titanium platform, also at the Emory University Genomics Facility.

### 16S Amplicon Library Preparation and Sequencing

Lake Lanier 16S rRNA gene amplicons were PCR-amplified from the same community DNA samples sequenced for metagenomic analysis using barcoded primers for the V1–V3 regions (Table S2 in File S1). Each 20 μl PCR mixture was comprised of 0.15 μl AccuPrime Taq DNA Polymerase High Fidelity (Invitrogen), 2 μl 10X AccuPrime PCR Buffer II, 13.85 μl nuclease-free water, 1 μl of 2 mM “B” adaptor-labeled 27F primer, 1 μl of 2 mM “A” adaptor-labeled and barcoded 534R primer, and 2 μl undiluted template DNA. PCR conditions consisted of 2 min incubation at 95°C followed by 25 cycles of 95°C, 20 sec; 50°C, 30 sec; and 72°C, 5 min. Three independent PCRs were carried out for each primer pair listed in Table S2 in File S1, the products of which were pooled for each sample. To further assess reproducibility and potential technical artifacts, we sequenced two separate amplicon pools from AUG1 (A and B) and three from NOV (A, B, and C; Table S3 in File S1). As an additional control sample, a mixture of DNA from four organisms grown in pure culture: *Escherichia coli* strain H1-sample ANK, *E. coli* str. K-12 substr. DH10B, and two environmental isolates: an *Enterococcus* sp. and a *Shewanella* sp., were also subject to 16S amplification and sequenced together with the Lake samples. PCR products were cleaned using Agencourt AMPure beads (Beckman Genomics). All seven samples were then pooled according to the Roche protocol into a mixture containing a final concentration of ∼10^7^ molecules/μl from each sample. The pooled amplicons were sequenced in duplicate (i.e., two halves of a plate) on the GS-FLX 454 Titanium instrument providing an average read length of 333.2 bp (Table S3 in File S1).

### 16S rRNA Gene Sequence Analysis

Each sample was separated from the run based on its barcode sequence using the Splitkeys.pl script from the AmpliconNoise package, version 1.2. The reads of each sample were then independently denoised to reduce sequencing and PCR single base substitutions and then chimera-checked using the PyroNoiseM + SeqNoise and Perseus shell scripts, respectively, in the AmpliconNoise package [6], [7]. A denoising shell script included a filter requiring a minimum flowgram length of 360 bp (including key and primer). To reintegrate all the samples for downstream comparisons, a combined fasta file of the denoised, chimera-checked sequences was created and the AmpliconNoise Qiime.pl script was used to make both a 3% and 1% OTU mapping file (i.e., OTUs were picked at both 97% and 99% identity). The QIIME software package, version 1.2.0 [30] was then used for 16S rRNA analysis, skipping OTU construction and beginning with the split_libraries.py (with –l 50–H 50) and pick_rep_set.py scripts using the pre-processed sequences. For many analyses, a subset of sequences obtained by randomly subsampling each dataset to the same depth (that of the smallest dataset) was used. OTUs that were identified in only one of the 14 datasets or that occurred as singletons were excluded from the analysis. Within QIIME, taxonomy was assigned with the RDP classifier based on a July 2011 version of the Greengenes reference OTU database [31] with the addition of a freshwater sequence database and taxonomy framework described in [32].

### Assembly and Phylogenetic Assignment of Metagenomic Reads

Metagenomic sequences from the same four timepoints were analyzed by creating a combined assembly from all four datasets after filtering the data for quality based on the Phred average per sliding window with Q≥20 and not allowing any N’s. The reads were assembled into contigs as described previously [29]; Table S4 in File S1 using the SOAPdenovo [33] and Velvet [34] pre-followed by assembly into longer contigs with Newbler 2.0. This hybrid protocol provided significantly longer contigs, with accuracy comparable with or higher than that of the contigs of Velvet or SOAPdenovo [35]. The resulting contigs were annotated using MeteGeneMark [36]. The predicted genes were subsequently searched against a database of all sequenced bacterial and archaeal genomes and their best match was used to infer the phylogenetic origin of contig sequences using the MyTaxa scheme developed in our lab ([37]; http://enve-omics.ce.gatech.edu/mytaxa/).

#### Nucleotide sequence accession numbers

16S datasets from the Lake Lanier samples were deposited in the Sequence Read Archive under the same projects as the previously submitted WGS datasets [29]: AUG1 (SRA029309.1), AUG2 (SRA029314.1), SEPT (SRA029315.1), and NOV (SRA029316.1).

## Results

### 16S rRNA Gene Amplicon Sequencing Reproducibility

We used the control mixture made up of 16S rRNA gene sequences from four organisms grown in isolation in the laboratory, to validate the denoising parameters and efficacy of OTU recovery and taxonomic assignments of amplicon sequencing. Following denoising and taxonomic binning of the sequences in this sample, four major (>1% of total sequences) 97% OTUs were identified in each of the two lane runs. The OTUs were identified using RDP taxonomy within QIIME at the order level as members of the *Enterobacteriales*, corresponding to the two *E. coli* strains of four control organisms used and accounting for about 50% of the reads, *Lactobacillales*, corresponding to the *Enterococcus* isolate used and accounting for about 20% of total, and *Alteromonadales*, corresponding to the *Shewanella* isolate and accounting for about 20% of the total. Thirteen additional OTUs with either singletons or few representative sequences (<0.1% of the total sequences) were also identified, but were likely the result of sequencing errors due to their low abundance and poor matches to the known, control sequences. These OTUs had taxonomic affiliations that matched the known control sequences at the phylum level, but most (9 of 13) could not be taxonomically assigned beyond that. Furthermore, the highly populated OTUs were the same between the two runs of the same sample, whereas the OTUs with a small number of sequences were not. We therefore determined that a reasonable filter would exclude OTUs with fewer than ten reads that were not found in both sequencing runs from the same sample, i.e., in both lanes 1 and 2. This filter, similar to what is used by others (e.g., [38]), was subsequently applied to our lake 16S rRNA gene datasets. Singletons were not removed for comparison with metagenomic sequences (see below).

The 14 datasets acquired from four temporally-distinct sampling events were processed using the standard QIIME protocol to bin reads into OTUs and then sub-sample each dataset to an even depth of 17,821 sequences, i.e., the number of sequences in the smallest dataset, in order to account for heterogeneity in the sequencing effort (Table S3 in File S1). As expected, slightly more OTUs were identified at the 99% level than the 97% level, but many of the additional 99% OTUs occurred as singletons (Fig. S2 in File S1). Each sample had ∼500 different OTUs at the 97% level and a total of 4,684 OTUs were identified. Only slight differences were detected in the numbers of OTUs recovered in independently sequenced datasets of the same DNA (Lane 1 vs. 2; e.g., for 571 and 520 OTUs for AUG1-1 and AUG1–2, respectively) or PCR replicates of the same water sample (replicates A and B, e.g., 545 and 487 OTUs for AUG1 A and AUG1 B, respectively), with the exception of NOV C which had notably fewer OTUs than NOV A-B (364 vs. 706 OTUs, respectively). Prior to the application of the aforementioned singleton filter, most (∼75%) of the OTUs were represented by only one sequence or were present in only one dataset, indicating that 16S-based sequencing approaches can overestimate OTU diversity within a taxonomic group in a sample and/or capture different members of the rare fraction. Excluding these OTUs resulted in a total of 1,067 OTUs present in at least one of the four samples and ∼250 OTUs specific to each dataset. Only ∼2% of the OTUs were present in all 14 datasets and ∼6% were found in all four timepoints, but these OTUs comprised ∼30% of the total reads.

**Figure 1:**
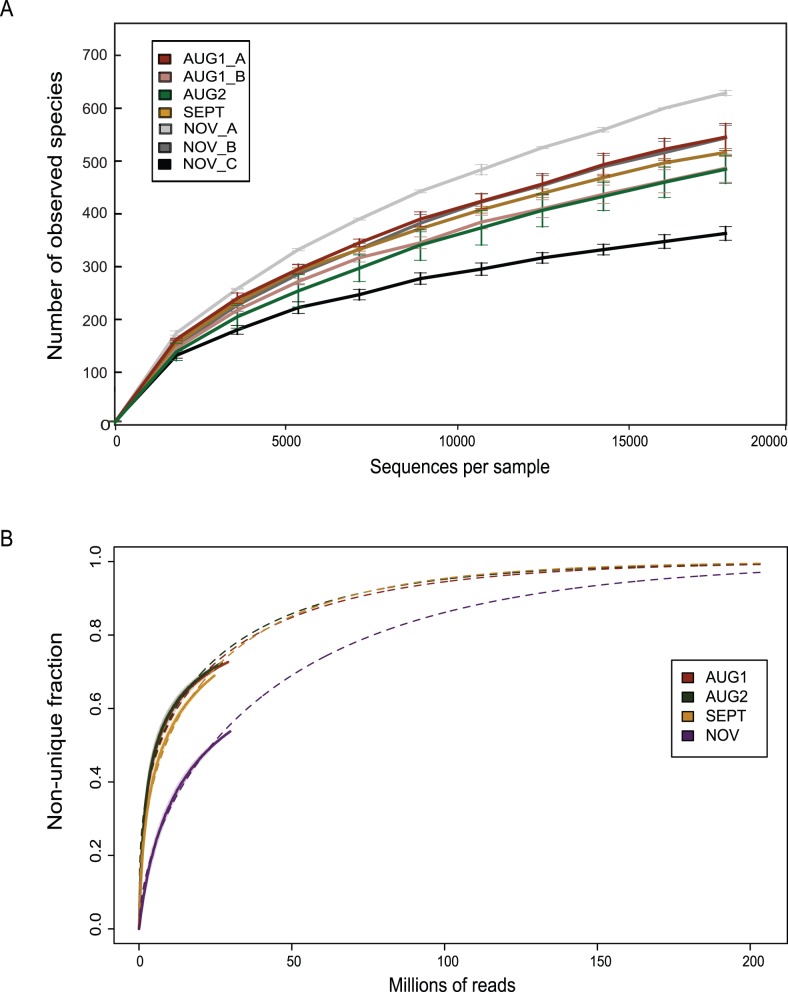
Diversity estimates for the four Lake Lanier timepoints . A) Alpha diversity based on observed species (97% OTUs) from 16S amplicons for each of the nine samples. Error bars represent the variation observed among duplicate sequencing runs. B) Redundancy curves of the metagenomes of the four timepoints using (see Methods for details). The curves show that NOV is a more diverse sample, e.g., with the same sequencing effort it results in a lower coverage.

Accordingly, observed species richness varied between samples. Rarefaction curves showed some of this variation among replicate samples (same DNA, independent PCR amplifications), particularly between NOV A/B (most diverse) and NOV C (least diverse) (Fig. 1A). The discrepancy in estimated diversity levels between the three November samples was surprising, given that the starting DNA for all three samples was the same. Nevertheless, the three NOV replicates were not significantly different from each other in terms of OTU composition, as evaluated by one-way ANOSIM [39]. All datasets shared more than half of their OTUs with both their corresponding technical sequencing replicate (i.e., lane 1 and lane 2) and their corresponding PCR replicate (e.g., A vs. B). Furthermore, when the datasets were filtered to remove singletons, the three NOV samples were highly similar to each other in terms of OTU composition relative to the samples from the other three timepoints (Fig. S3 in File S1). Good’s nonparametric coverage estimator [40] was similar among NOV A/B (98.2 and 98.5, respectively) and slightly higher for NOV C (99.2). Because the original, non-rarefied NOV C datasets contained the fewest sequenced reads of all the 14 datasets (Table S3 in File S1), the difference between NOV C and NOV A/B could be related to the sizes of the original datasets (Table S3 in File S1), and the number of singletons captured in the datasets (resulting from sequencing or PCR amplification artifacts) as opposed to the presence of entirely different OTUs.

There was no significant difference (G test of independence) in the presence/absence of OTUs at the 97% level between either the sequencing replicates or the seven datasets representing the various replicates from the four timepoints. However, there was distinct partitioning of OTUs between samples in terms of relative abundance of shared OTUs, with few differences between sequencing replicates (Fig. S3 in File S1). Additionally, OTU composition of AUG1, AUG2, and SEPT clustered uniquely from the NOV samples (Fig. S3 in File S1). This was verified when biological replicates were combined and the four timepoints were compared, revealing that the NOV OTU composition, phylogeny (evaluated with weighted UniFrac distance), and abundance was significantly different from the other three timepoints (One-way ANOSIM, p<0.01). In general, abundant taxa were common between the four timepoints.

### 16S rRNA Gene-based Community Composition, Diversity, and Dynamics between Four Timepoints

**Figure 2:**
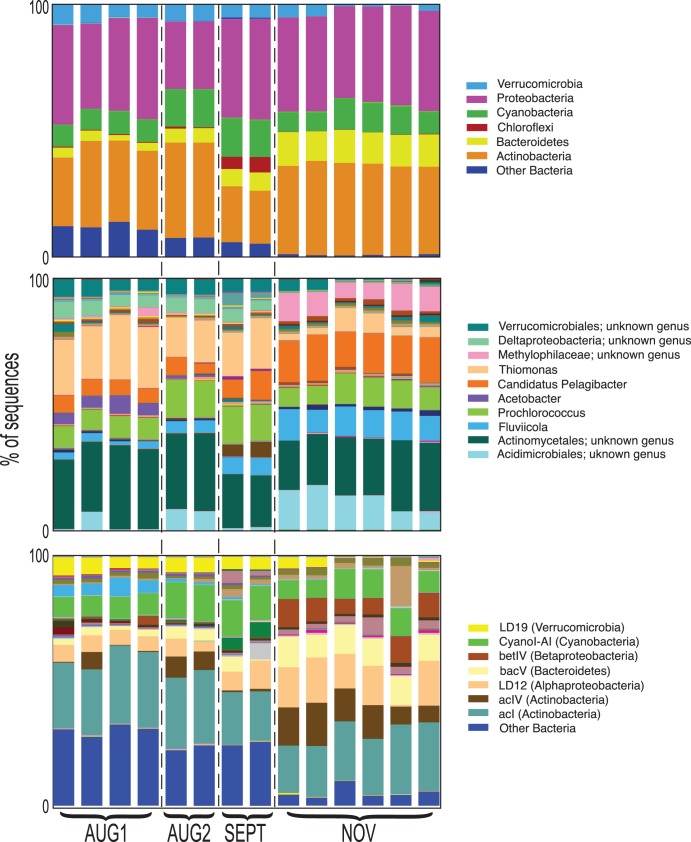
Community composition shifts over time as revealed by 16S data. Taxonomic binning of 16S amplicon sequences for each of the 14 individual datasets at the phylum (top) and genus (middle) levels were based on the July 2011 version of the Greengenes database [31]. Freshwater lineages (bottom) were based on a freshwater database according to the taxonomy framework described in Newton et al., 2011. Datasets are ordered left to right by date, technical sequencing replicate (lane 1 and lane 2), and DNA replicate (A, B and C). Taxa identified as major drivers of the differences between timepoints (SIMPER analysis) are labeled (see figure key).

Most major bacterial phyla were present in Lake Lanier and there was a high representation of common freshwater taxa [32] such as *Actinobacteria*, *Cyanobacteria*, *Verrucomicrobia* and *Proteobacteria* (particularly *Betaproteobacteria*). In fact, *Proteobacteria* and *Actinobacteria* were the most frequently observed, with nearly 20–40% of the total sequences identified as members of these phyla at each timepoint (Fig. 2, top). About 10% of the OTUs from all lake datasets matched previously sequenced organisms with high sequence identity (>95%), e.g., *Synechococcus sp.* and *Polynucleobacter necessarius*, a betaproteobacterium frequently detected in freshwater [41]. More divergent sequences (e.g., 80–85% sequence identity) were also observed, indicative of uncharacterized taxa more distantly related to well-characterized lineages, although it is also possible that some of these divergent sequences could arise from sequencing errors (see below). Nearly 35% of the OTUs were highly (>95% identity) similar to known freshwater 16S rRNA sequences, including 118 OTUs with reads 100% identical to freshwater tribes (a taxonomic level below clade defined in [32]) belonging to *Actinobacteria, Alpha-, Beta- and Gamma-proteobacteria, Bacteroidetes,* and *Verrucomicrobia*.

The OTUs in each of the combined datasets from the four timepoints were binned into phyla, genera, and described freshwater lineages to better characterize the temporal shifts in a taxonomic context. Of 17,821 sequences in each library, ∼90% could be assigned to a known phylum and between 32–47%, depending on the dataset, to a known genus in the Greengenes database. In addition to the differences in the 97% OTU composition noted above, the NOV sample was significantly different from the others in terms of phyla, genera, and clade (ANOSIM with Bray-Curtis metric, p<0.01; Fig. 2). The differences, however, were due more to variations in relative abundances of specific phyla or genera than to differences in the presence or absence of taxonomic groups at these levels (as assessed by the G-test of independence), although phylogenetic differences were found at a finer-scale resolution (as assessed by UPGMA analysis and tribe-level comparisons). Thus, the same major phyla and genera were typically found in all four timepoints, but the abundances of these groups varied, as did individuals comprising these groups. Similarity Percentage (SIMPER) analysis [39] identified specific taxa as primary drivers of the differences between samples (Fig. 2). Much of the difference (6%) between AUG1 and NOV was attributed to OTUs that could not be classified at the phylum level (“Other Bacteria” in Fig. 2). Generally, NOV had a lower relative abundance of *Verrucomicrobia* and a higher relative abundance of *Bacteroidetes* compared to the first three samples. *Cyanobacteria* abundance increased following the storm event (AUG2) but returned to AUG1 levels (∼8% of the total phyla) in NOV.

While variation at the genus level was more difficult to assess due the challenge of assigning genera with high confidence, we were able to identify a number of known genera and their freshwater taxonomic affiliations (based on the lineages described in [32]) with significantly different relative abundances between the four timepoints (Fig. 2). For example, *Thiomonas* comprised 16–23% of the genera in AUG1-SEPT, but less than 5% of the genera in NOV. *Pelagibacter* increased in relative abundance from ∼6% in AUG1–SEPT to ∼16% in NOV. This genus corresponded to LD12 tribe (alfV-A) [32], the freshwater sibling to the marine SAR11 group (Fig. 2, bottom), which also increased in abundance in NOV. Other genera such as *Prochlorococcus*-like sequences varied between the four timepoints, and was highest in AUG2 (15%) following the strong summer storm and lowest in AUG1 (8%). Interestingly, *Prochlorococcus* is a ubiquitous marine organism [42], but *Prochlorococcus*-like organisms have seldom been identified in freshwater systems [43], [44]. We suspect, however, that these sequences were misclassified when using the RDP taxonomy, as evidenced by the high representation of *Synechococcus* among both the 16S rRNA gene amplicons re-analyzed using NCBI taxonomy and the metagenome contigs in all four timepoints (see below), as well as the known abundance of *Synechococcus* in freshwater lakes [32]. These *Prochlorococcus*-like sequences were also assigned to the *Cyanothece* related freshwater CyanI-A1. This illustrates one of the difficulties in using 16S rRNA gene sequences to examine populations at the genus level or lower.

Among the *Actinomycetales* that could not be assigned to a known genus, we examined the freshwater lineages that they associated with. The acI lineage [32] dominated all four sampling points, typically representing 20–30% of the identified lineages. Among the tribes affiliated with this lineage, acI-A6 and acI-C2 dominated all samples, but showed different abundance profiles: acI-A6 was roughly stable over time, accounting for 15–20% of the identified lineages while acI-C2 peaked after the storm at AUG2 to about twice the levels seen at the other timepoints. Other acI tribes such as acI-B1 were more abundant in the NOV sample and acI-A1 was most abundant in AUG1. The second most abundant *Actinobacteria* lineage, acIV, also showed variation in the abundance profiles of the associated clades, with some increasing in abundance in AUG2 and NOV (acIV-A), and others being nearly absent in AUG1–SEPT but peaking in NOV (acIV-B and acIV-C). Overall, substantial variation was seen among individual clades and tribes comprising the identified lineages. Because there are so few defined lake species and genera, these phylogenetic classifications cannot be gleaned from the genus level assignments and individual OTUs do not always provide detailed taxonomic information. Thus, comparisons to well-described, relevant 16S rRNA gene databases such are useful when available.

### Comparisons to Corresponding Metagenomes

We compared the 16S rRNA gene findings to those from companion metagenomes, first by estimating the diversity of each metagenomic dataset using a new method developed in our group ([45]; http://enve-omics.ce.gatech.edu/nonpareil/) that determines the relative complexity of a metagenomic dataset using the extent of redundancy of its reads. From these estimates, the AUG1, AUG2 and SEPT assembled contig sequences had on average 2–5X coverage, while the NOV coverage was ∼0.5X and required significantly higher sequencing effort in order to achieve nearly complete community coverage (Fig. 1B). These findings agreed well with our 16S rRNA gene observations of increased diversity in sample NOV A relative to the other timepoints and different from NOV B and C (Fig. 1A). Thus, the metagenomic analysis indicated that the NOV A 16S datasets were likely the most representative of the three NOV replicates.

A combined assembly from the four metagenomic datasets was then used to obtain reference contigs, representing distinct populations, and follow their abundance over time. Unlike the 16S amplicon-based OTUs, more than 90% of which were unique to one or more timepoints, 79% of the 217,149 contigs longer than 500 bp, were detected at all four timepoints. Despite the differences in estimated diversity between the four metagenomic samples (read redundancy, Fig. 1B), the differences in terms of the composition and abundance of different contigs were not significant (ANOSIM with Bray-Curtis metric, p<0.01). These findings indicated that, unlike individual reads (read redundancy), which can represent low-abundance in addition to high-abundance community members, long contigs, which typically represent abundant community members, were found with roughly similar coverage levels in the four timepoints. Additional diversity that may have been present among shorter contigs in the metagenome (<500 bp) was not assessed but likely differed between the samples, similar to the read data above.

**Figure 3:**
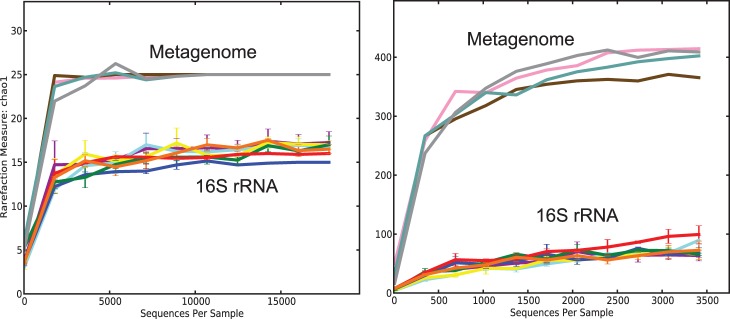
Sequence diversity of the samples used in this study . Chao1 diversity estimates of datasets based on phylum (A) and genus (B) level taxonomic classification are shown for all four metagenomic timepoints and seven selected 16S amplicon datasets.

The organisms identified from 16S rRNA gene sequencing were compared to those identified from the metagenomes. The OTUs in each amplicon dataset were reanalyzed using NCBI taxonomy both with and without singleton and other sequence–removal filters (which reduced the number of identified OTUs by ∼10%) for consistency between the 16S and whole-genome. To determine the taxonomic origin of short-read metagenomic sequences, we used MyTaxa, an advanced taxonomic classifier developed in our group that combines homology- and phylogenetic-based approaches to assign putative taxonomic origin to assembled contigs [37]. Contig sequencing depth (reads/length) was used as a proxy for taxon abundance. With this approach, 1.5 times and ∼10 times as many phyla and genera, respectively, were identified within the metagenomic contigs than the 16S amplicons (based on OTUs found in the database), regardless of whether or not the singleton filter was applied to the amplicon sequences. ChaoI diversity estimates based on phylum- and genus-level assignments were therefore different between the metagenomic and 16S amplicon datasets (Fig. 3).

**Figure 4:**
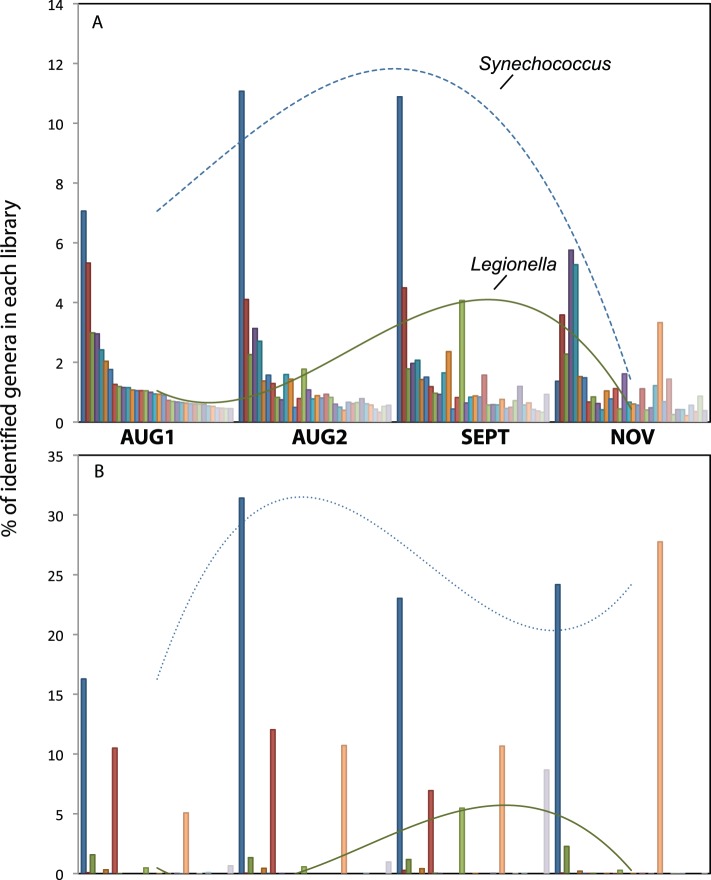
Individual genera abundance shifts over time based on 16S and metagenomes. Genus-level taxonomic trends for a subset of genera identified within the metagenomic contigs (A) and 16S rRNA amplicon (B) datasets, based on NCBI taxonomy, are shown. The lines represent the general temporal trends of two genera, *Synechococcus* and *Legionella*, in each dataset.

The taxonomic composition of each 16S rRNA gene library was generally similar to its corresponding metagenome at the phylum level, although some phyla were more represented in the metagenome analysis than the 16S amplicon analysis, including *Firmicutes* and *Planctomycetes*, while others such as *Cyanobacteria* were more abundant among the 16S rRNA gene amplicons (Fig. S4 in File S1). A few phyla were only found in the metagenomic contigs, albeit in low abundances (<0.2% of the total number of phylum-assigned contigs), and included the *Dictyoglomi, Fusobacteria, Synergistetes,* and *Deinococcus-Thermus*. At the genus level, however, there was a large amount of variation between the metagenomic contigs and the 16S rRNA sequences. Some genera showed similar trends and relative abundances over time (e.g., *Legionella*; Fig. 4) while others showed substantial differences (e.g., *Burkholderia* and *Synechococcus*; Fig. 4). *Thiomonas* accounted for ∼45% of the 16S amplicons identified at the genus level in AUG1 (and 23% of all OTUs using RDP taxonomy, Fig. 2), but only ∼0.3% of the metagenome contigs identified at that level. Although the amplicon approach appears to overestimate the abundance of this group (see below), the high abundance of this genus is probably not wholly a PCR artifact, as almost 10% of the partial 16S rRNA gene sequences recovered in the metagenome (see below) were 100% identical to the 16S rRNA gene amplicon sequences assigned to *Thiomonas*. Part of the discrepancy between the contig and 16S rRNA gene amplicon assignments could instead be due to the fact that there were only two complete *Thiomonas* reference genomes in the database but 243 distinct *Thiomonas* OTUs in the Greengenes OTU database at the time of analysis.

As noted above, the genus level may frequently mask important levels of intra-genus population differentiation and heterogeneity, which typically remain inaccessible to short-read, 16S rRNA gene-based analysis that target a single variable region of the 16S rRNA gene [46], [47]. This was evident following a more detailed investigation of three different genera, *Synechococcus, Burkholderia,* and *Legionella,* whose relative abundance profiles over time varied between the 16S rRNA gene amplicons and the metagenome. Each of these genera was represented in the 16S amplicon data by several different OTUs (*Synechococcus,* 80; *Burkholderia,* 18; and *Legionella,* 56) and hundreds to thousands of different contigs in the metagenomic datasets. The contigs assigned to each genus with our MyTaxa algorithm were clustered using the Pearson correlation metric in order to collapse the contigs into populations with similar abundance profiles over the four timepoints, likely representing similar populations [48]. Often, the two methods were congruent, but this analysis revealed variations not captured when the data were assessed at the genus level. For example, *Synechocococcus* was dominated by a single 16S rRNA gene OTU for the first three timepoints, which was replaced by two different OTUs in the NOV sample that were either undetected or present in low quantities at earlier timepoints, keeping the total abundance of *Synechococcus* in the four samples relatively constant (Fig. S5 in File S1). Although similar trends were observed among the metagenome contigs, the contigs that increased in abundance in the NOV sample relative to the other three were present at such low levels that the overall percentage of *Synechococcus* in the NOV sample was low, making the overall patterns observed for this genus different between the two methods. It should also be noted that, in contrast to *Thiomonas*, there are many *Synechococcus* reference genomes. Populations (metagenomes) or OTUs (16S rRNA gene data) within the other two genera examined generally displayed a similar trend as well, i.e., several abundant taxa were observed in the AUG1 to SEPT samples that were apparently replaced by other taxa in NOV (Fig. S5 in File S1). Overall, the metagenomic contigs and the 16S rRNA gene sequence data generally agreed with each other, but the relative abundances of the individual taxa within the genera contributed to seemingly large differences.

We also compared partial 16S sequences recovered in a shotgun 454 metagenome generated from the AUG1 sample [29] to the PCR-based identification of 16S rRNA gene amplicon sequences. Among the 558 partial 16S sequences captured in the AUG1 454 metagenome, there were 302 different OTUs, approximately half as many as in the PCR-based dataset, which had more than 30,000 individual 16S rRNA gene amplicon sequences (Fig. S2 in File S1). However, subsampling the 16S rRNA gene amplicon sequences to the same depth as the 16S rRNA gene reads from the metagenome, i.e., 558, yielded only about 80 different OTUs in each 16S rDNA amplicon dataset, indicating that the metagenome provided more information on community composition at the per-read level, but the number of reads obtained from the 16S rDNA amplicon sequencing approach offset this inequality. Some phyla such as TM7 were captured in the PCR-based datasets, albeit in low abundance (<0.1%), but were not found among the shotgun-derived 16S sequences, probably because of the lower number of sequences, while others such as *Chlamydiae* were only found within the shotgun 16S rRNA gene sequences, perhaps because of primer/PCR biases in the amplicon approach. The relative number of OTUs attributed to *Actinobacteria* and *Verrucomicrobia* was higher among the shotgun sequences than the PCR-based sequences and corresponded well with the lineages identified from comparison to the freshwater 16S database (above), indicating that the shotgun library is sometimes able to capture more taxonomic diversity than 16S amplicons. Our observations corroborate those seen by others that the 16S rRNA gene sequences derived from metagenomic datasets vs. PCR amplification are roughly similar at broad taxonomic classifications (e.g., [49]), but that the number of OTUs identified by 16S rRNA gene sequencing is larger simply by virtue of the number of sequences obtained and that shotgun approaches capture greater diversity due to the lack of PCR primer specificity [50].

Finally, to gain further insight into individual populations, full-length 16S sequences were reconstructed separately from each of the AUG1–SEPT metagenomes [51] and were compared to the 16S rDNA amplicon OTUs in terms of sequence similarity and abundance patterns over the timepoints. One full-length reconstructed OTU most likely corresponding to 16S amplicon “OTU 2” (represented by sequence GKJT1QE01CC3RV) was identified as a member of the order *Burkholderiales*, had no known close relative at the genus level, and comprised 16–20% of the 16S rRNA genes among the fragments captured in the three metagenomes. This sequence was named “FJ820419” for its closest relative in the Silva 16S database. OTU 2 and FJ820419 were assigned to different families within the *Burkholderiales*: OTU 2 to *Alcaligenaceae* and FJ820419 to *Burkholderiaceae*. Although they both had >98% identity to a known freshwater *Betaproteobacteria* named LakTan18, neither could be assigned to taxa lower than the family level; the closest genus match of OTU 2, with ∼94% identity, was to either *Thiomonas* or other uncultured *Burkholderiales*. In fact, OTU 2 was the biggest contributor to the *Thiomonas* sequences identified among the 16S rDNA amplicon OTUs. Potential biases in the amplification of the 16S rRNA gene and the short length of the 16S amplicon reads as well as the predictive nature of the full length 16S rRNA genes sequence reconstruction from the metagenomes confounded our ability to accurately link the two methods to a single source organism. Nevertheless, the relative abundances of FJ820419 in the four timepoints gauged by recruitment of reads to the full-length FJ820419 sequence were comparable to the abundances of OTU 2 in each amplicon datasets (e.g., 22% vs. 28%; Fig. S6 in File S1), providing further evidence that the metagenome and 16S rDNA amplicon sequencing can sometimes identify the same populations, but the taxonomic assignments are not always consistent.

## Discussion

### Sequencing Replicates are more Consistent than Sample Preparation Replicates

While investigating temporal dynamics and response to potential disturbances in a freshwater, mesotrophic lake community, we employed numerous methodological and experimental replicates, providing a means to comprehensively evaluate specific limitations of some of the commonly used methods for microbial community characterization. Although denoising and chimera checking can reduce the number of potentially spurious sequences arising from both PCR and sequencing errors [7], such processing cannot eliminate all biases, as evidenced by the identification of thirteen different OTUs in a control DNA sample generated from only four different organisms. The filter that we used here, i.e., removing singletons and OTUs present in only one of our replicate datasets, has the disadvantage of confounding our ability to estimate diversity and identify rare members of the community. In agreement with other recent studies [13], [52], it is clear that the inclusion of “synthetic communities” is highly advantageous for 16S amplicon sequencing in order to determine biases of sequencing runs, optimal filtering, and assess samples properly. Further, the use of replicate samples enabled us to better pinpoint both advantages and limitations of the 16S rDNA amplicon sequencing approach. We observed differences in the number of OTUs and, therefore, the projected diversity, in replicate datasets obtained from the same template DNA but from different pools of PCR amplicons. Because replicate sequencing runs (i.e., lane 1 vs. lane 2) were similar and the differences between the NOV triplicates correlated with differences in the read yield and singletons observed in each dataset, it appears that the discrepancies in diversity estimates are likely introduced either at the PCR and library preparation steps or sequencing steps, which are virtually impossible to control even when all libraries are constructed in the same manner. Specifically, among the three NOV samples, the smallest dataset (NOV C) had the fewest number of singletons and the lowest estimated diversity and the resulting biases were not fully eliminated by sub-sampling to an even sequencing depth prior to analysis. It is difficult to discern whether the differences between NOV A/B and NOV C arise from errors at the PCR step or from the lower yield of reads (and whatever underlying cause resulted in fewer reads at the sequencing step), but it is clear that replicate, at least triplicate, biological samples should be prepared for 16S rDNA amplicon sequencing from any sample, even if multiple PCRs are pooled for a single sample. Replicate libraries from each pooled PCR sample should be independently sequenced as well, which is increasingly feasible given the advances and cost effectiveness of new sequencing technologies. Nevertheless, the differences between sample replicates (both lane 1 vs. lane 2 as well as “A, B, and C” PCR replicates) were small relative to the differences between the four timepoints, at least in terms of community composition (e.g., Fig. S3 in File S1). Additionally, although differences in diversity estimates between NOV A, B, and C were evident (Fig. 1A), the NOV samples were clearly more similar to each other in terms of OTU composition and distinct from the AUG and SEPT samples (Fig. S3 in File S1). Therefore, the types of replicates included in a study should be carefully considered in the context of the comparisons being made as well as the sequencing platform being used [53].

### The 16S rRNA Gene Identifies Broad Levels of Community Composition While Metagenomics Captures a Higher Level of Diversity

The assignment of taxonomic origin to metagenomic sequences continues to be a hurdle and the confidence with which 16S rRNA gene sequences can be assigned to deep taxonomic levels such as genus can be low. Classification of both protein-encoding and 16S sequences is also limited by the databases used for sequence comparisons; although there are several high quality, comprehensive and curated 16S databases compared to genomic databases, they are still limited, as evidenced by the paucity of 16S rRNA gene reads that could confidently be assigned to a genus and the increased resolution obtained by comparison to a relevant, well-curated freshwater database. Many databases are biased in their compositions; e.g., ∼30% of the Greengenes database are *Proteobacteria,* which comprise ∼5% of the database of complete microbial genomes used to assign taxonomic affiliations to metagenomic contigs. We showed that when the genome database is limited, the metagenome data can miss taxa due to sequences being unassigned, while in cases where there are ample reference genomes and gene sequences, genus-level assignments of the 16S rRNA gene amplicons can be less reliable than those of the contigs, likely due to the region of the 16S rRNA gene chosen [54], [55] and the high conservation of the 16S rRNA gene, which can mask important level of micro-heterogeneity. By assigning taxonomic origin to metagenomic sequences, we were able to get a more detailed sense of the community structure than by 16S rRNA gene sequencing alone, but the confidence with which we can make these assignments remains a challenge. As the databases expand, so does our ability to more accurately assign taxonomy to reads.

Comparing the metagenomic and 16S rRNA amplicon sequences revealed some notable patterns. Many more phyla and genera were identified among the metagenomic than the amplicon sequences, a likely consequence of both of the databases used and biases in PCR amplification and amplicon sequencing. The latter biases can produce differences in estimated diversity levels depending on the sequencing platform, the discriminatory power of the region of the 16S rRNA molecule targeted [53], and the fact that some taxa such as *Planctomycetes,* which were seen in higher abundance in the metagenomes (Fig. S4 in File S1), are detected less efficiently, or not at all, by some 16S rRNA primer sets [56]. Groups such as *Proteobacteria,* which are represented well in the 16S and genome databases, showed similar abundances between the two approaches. Binning either the contigs or the 16S rDNA amplicons into taxonomic groups at finer-scale resolution (i.e., genus level or species level) provided vastly different pictures of community composition for many, but not all, taxa. We also demonstrated that evaluating genus-level trends masks the variation and diversity of individual “species” or OTUs/genotypes within any genus, which can be partially overcome by binning both metagenomic contigs and 16S rRNA gene sequences into OTU-like units based on recruitment of reads to contigs and percent identities, respectively.

It is clear from our analysis that different patterns arise for different groups of organisms depending on the analysis method used. Metagenomic contigs, 16S rRNA gene sequences encoded in metagenomic data, and 16S rDNA amplicon sequences were sometimes concurrent, especially for taxa that were both abundant in the sample and well-represented in 16S rRNA and genome databases, while other times they provided a vastly different picture of microbial community composition and dynamics. For example, *Synechococcus* populations, one of the most abundant genera detected by both approaches, were tracked with higher resolution, both in terms of number of distinct OTUs present as well as OTU abundance patterns over time, by metagenomics (Fig. S5 in File S1). The 16S rDNA amplicon approach clearly has the potential to artificially increase the perceived diversity of a sample more so than the metagenome due to errors or artifacts, as evidenced by our examination of a mixture of known 16S rRNA gene sequences. As long as sufficient reference genomes exist for identification, the metagenome performs well in describing the taxonomic composition of a sample. In addition, metagenomics offers the potential to investigate 16S rRNA gene fragments recovered in metagenomic reads without amplification as well as robust taxonomic assignment of contigs, description of genomic populations based on contig dynamics, and even the reconstruction of full-length 16S rRNA gene sequences.

### Temporal Changes in Microbial Community Composition

In evaluating microbial communities, it is informative to not only quantify the relative abundances of different taxa, but also to track these abundances over time and following potential perturbations such as heavy rainfall or lake turnover. We are particularly interested in such dynamics in Lake Lanier due to its regional significance and the general lack of information on microbial communities in southern temperate lakes. Taking the above limitations in assigning taxonomic affiliations to both 16S rDNA amplicon and metagenomic sequences into account, we observed members of several organisms that showed different patterns of abundance across the four samples taken on short-term timescale. The microbial communities from Lake Lanier experience broad shifts in community diversity over the course of several months, but the community changed little over the course of 3 days, despite the occurrence of a strong summer storm between the first two sampling timepoints. The resistance of the microbial community to the pulse disturbance of high precipitation was somewhat surprising, given that many microbial communities are sensitive to ecosystem-level disturbances [57]. However, our observations combined with the fact that there was no discernable difference in the basic water chemistry before and after the storm (Table S1 in File S1) indicate that either such a rainfall event was not a chemical or thermal disturbance or that the system recovered more rapidly than our sampling scheme could detect. In contrast, the community changed more substantially between August and November, correlating to *in situ c*onditions in the lake such as the transition from stratified to well-mixed (Fig. S1 in File S1; Table S1 in File S1), consistent with previous observations that bacterioplakton often experience seasonal shifts in both lakes [58]–[60] and marine environments [2]. Thus, short-term, pulse disturbances related to a meteorological event sometimes have less of an effect on the microbial communities than anticipated while long-term disturbances occurring that occur during lake turnover can cause significant shifts in microbial community composition. The general trends for the microbial community diversity observed from metagenomic reads and 16S rDNA amplicon reads were comparable: both approaches showed similar diversity levels in AUG1, AUG2, and SEPT and that the NOV sample was relatively more diverse. Mixing events have previously been identified as disturbances that can dramatically shift microbial community composition [61], [62]. When mixing events were performed in experimental manipulations, changes in community composition were presumed to be due to the introduction of nutrients from the hypolimnion [61]. Similar to our observations in the NOV timepoint, epilimnion samples in experimental manipulations were also shown to increase in richness following lake mixing [62]. Using a combination of metagenomics to track contigs and 16S rRNA gene sequencing to track OTUs between the four temporally separated samples from Lake Lanier, we were also able to identify some general patterns in bacterioplankton community composition, although the specific dynamics sometimes differed between the two methods, as noted above. The changes in community composition in Lake Lanier between the first three timepoints and the fall mixing sometimes echoed shifts in individual taxa seen in very different northern lakes that experience ice cover; for example, both LD12, the freshwater *Pelagibacter* relative, and a member of the acIV lineage increased in abundance in NOV in Lake Lanier and also peaked in autumn in a Swedish lake [25]. In a North Sparkling Bog in the northern US, there was a slight increase in *Gammaproteobacteria* post-mixing, similar to in Lake Lanier [62]. Despite these similarities, we observed many taxa whose abundances over time did not correspond with those in these northern lakes, indicating that bacterioplankton in southern temperate lakes such as Lake Lanier might behave differently in response to similar seasonal changes than their relatives in northern lakes.

This study provides insights into the Lake Lanier planktonic microbial community and advances the approaches for assessing microbial community diversity and dynamics *in situ*. A combined approach using both metagenomics and 16S rRNA gene sequences can help provide a complete picture, but sequencing controls and replicates are advised, especially when trying to infer diversity levels. Using 16S rRNA gene fragments recovered in a shotgun metagenome has the benefit of removing the initial PCR amplicon biases and providing a means to do both functional genomics and 16S analysis with the same sequence dataset. Here, we demonstrated that Lake Lanier microbial communities are resistant to a short-term rainfall disturbance in the summer, but shift in composition and diversity during the fall mixing, although these shifts do not always correspond to those seen in other, well-studied freshwater lakes. Continued, long-term seasonal characterizations of Lake Lanier will help validate the patterns observed here.

## Supporting Information

File S1 **The file includes Figures S1 to S6 and Table S1 to S4.** Figure S1 Dissolved oxygen and temperature profiles for July–December 2009 at the Brown’s Bridge location of Lake Lanier. Figure S2 Number of OTUs identified in each dataset. Figure S3 Similarity of datasets based on shared OTUs. Figure S4 Differences in phylum abundance based on 16S and metagenomes. Figure S5 Differences in abundance of individual genera based on 16S and metagenomes. Figure S6 Abundance of an individual *Burkholderia*-like population based on 16S amplicon vs. 16S metagenomic reads. Table S1 Characteristics of Lake Lanier at Brown’s Bridge for several dates in 2009. Table S2 V1, V3 specific primers used for amplification of the 16S rRNA gene. Table S3 16S rRNA gene amplicon library statistics. Table S4 metagenome library statistics.
(PDF)
