# Supplementary material for: Strengths and Limitations of 16S rRNA Gene Amplicon Sequencing in Revealing Temporal Microbial Community Dynamics
Source: PLoS One. 2014 Apr 8;9(4):e93827. doi: 10.1371/journal.pone.0093827 (PMC3979728; doi:10.1371/journal.pone.0093827)
Supplement: File S1 — The file includes Figures S1 to S6 and Table S1 to S4. Figure S1 Dissolved oxygen and temperature profiles for July–December 2009 at the Brown’s Bridge location of Lake Lanier. Figure S2 Number of OTUs identified in each dataset. Figure S3 Similarity of datasets based on shared OTUs. Figure S4 Differences in phylum abundance based on 16S and metagenomes. Figure S5 Differences in abundance of individual genera based on 16S and metagenomes. Figure S6 Abundance of an individual Burkholderia-like population based on 16S amplicon vs. 16S metagenomic reads. Table S1 Characteristics of Lake Lanier at Brown’s Bridge for several dates in 2009. Table S2 V1, V3 specific primers used for amplification of the 16S rRNA gene. Table S3 16S rRNA gene amplicon library statistics. Table S4 metagenome library statistics. (PDF) [file pone.0093827.s001.pdf]

TABLE S1. Characteristics of Lake Lanier at Brown's Bridge for several dates in 2009.

Date	Temp (°C)	pH	Turbidity (NTU)	Dissolve d solids (g/L)	Conductivity (μS/cm)	Dissolved O_2 (mg/L)	NH4 (mg/L)	NO2+NO3 (mg/L)	Total N (mg/L)	Total P (mg/L)	Chl a (μg/L)	Total Organic Carbon (mg/L)
20-Aug-2009[b]	28.9	7.86	-	-	49.4	7.57	0.03	0.04	0.28	0.03	5.28	<1.0
26-Aug-2009[a]	28.5	7.61	222	0.03	50	7.9	-	-	-	-	-	-
28-Aug-2009[a]	28.5	7.71	230	0.03	50	7.8	-	-	-	-	-	-
7-Sep-2009[a]	-	-	-	-	-	-	-	-	-	-	-	-
23-Sep-2009[b]	24.1	6.75	-	-	44	7.1	0.03	0.02	0.22	0.04	5.88	1.8
8-Nov-2009[a]	19.2	6.78	3.2	0.03	46	7.3	-	-	-	-	-	-
14-Nov-2009[b]	17	7.28	1.7	-	41	9.3	0.01	0.10	0.72	0.04	7.68	2.1

[a] Samples corresponding to the 16S rRNA amplicon and shotgun metagenome sequencing datasets

[b] Data obtained from the Georgia Department of Natural Resources

Table S2. V1-V3 specific primers used for amplification of the 16S rRNA gene. Lowercase indicates the adaptor sequence and bold indicates the sample-specific barcode (multiplex identifier; MID).

Primer	Sample	Sequence
27F	All	5'-cctatcccctgtgtgccttggcagtctcagAGAGTTTGATCCTGGCTCAG-3'
534R	AUG1-A	5'-ccatctcatccctgcgtgtctccgactcag**AGACAC**ATTACCGCGGCTGCTGG-3'
	AUG1-B	5'-ccatctcatccctgcgtgtctccgactcag**CGACTC**ATTACCGCGGCTGCTGG-3'
	AUG2	5'-ccatctcatccctgcgtgtctccgactcag**ACTCAC**ATTACCGCGGCTGCTGG-3'
	SEPT	5'-ccatctcatccctgcgtgtctccgactcag**ACGCGC**ATTACCGCGGCTGCTGG-3'
	NOV-A	5'-ccatctcatccctgcgtgtctccgactcag**CCTCTC**ATTACCGCGGCTGCTGG-3'
	NOV-B	5'-ccatctcatccctgcgtgtctccgactcag**AGCTTC**ATTACCGCGGCTGCTGG-3'
	NOV-C	5'-ccatctcatccctgcgtgtctccgactcag**AAGCCGC**ATTACCGCGGCTGCTGG-3'

TABLE S3. 16S rRNA gene amplicon library statistics.

Sample	Date	No. sequences Lane 1	No. sequences Lane 2
AUG1 A	26 Aug 2009	38,582	38,974
AUG1 B	26 Aug 2009	34,183	34,908
AUG_2	28 Aug 2009	44,405	44,417
SEPT	07 Sep 2009	34,004	34,439
NOV A	08 Nov 2009	38,361	39,486
NOV B	08 Nov 2009	58,427	60,847
NOV C	08 Nov 2009	29,288	30,094
Control mixture	----	13,591	12,701

TABLE S4. Metagenome library statistics. The individual metagenomes were described previously (1)

Sample	Date	No. of reads	Avg read length (bp)	Total no. contigs	Total length of contigs (Mb)
AUG_1	26 Aug 2009	23,820,047	100	168,215	93.08
AUG_2	28 Aug 2009	23,501,825	100	320,279	143.12
SEPT	07 Sep 2009	21,591,982	100	270,874	120.39
NOV	08 Nov 2009	24,579,678	100	380,549	130.87
Consensus	--	--	100	217,149	269.34

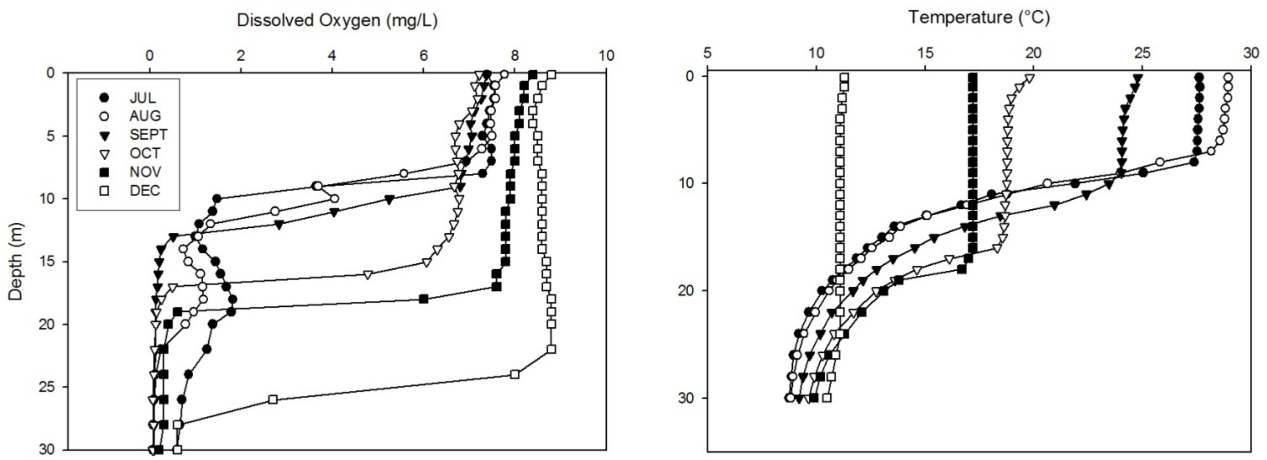

Figure S1. Dissolved oxygen (left) and temperature (right) profiles for July-December 2009 at the Brown's Bridge location of Lake Lanier. Lake Lanier stratifies during the early spring and summer and is strongly stratified in the summer. It begins mixing in the late fall/early winter and becomes well mixed in the winter. Samples for 16S rRNA and metagenomic characterization were collected at a depth of 5 m, within the epilimnion. Lake Lanier profile data was obtained from the Georgia Department of Natural Resources from a single sampling day in each month.

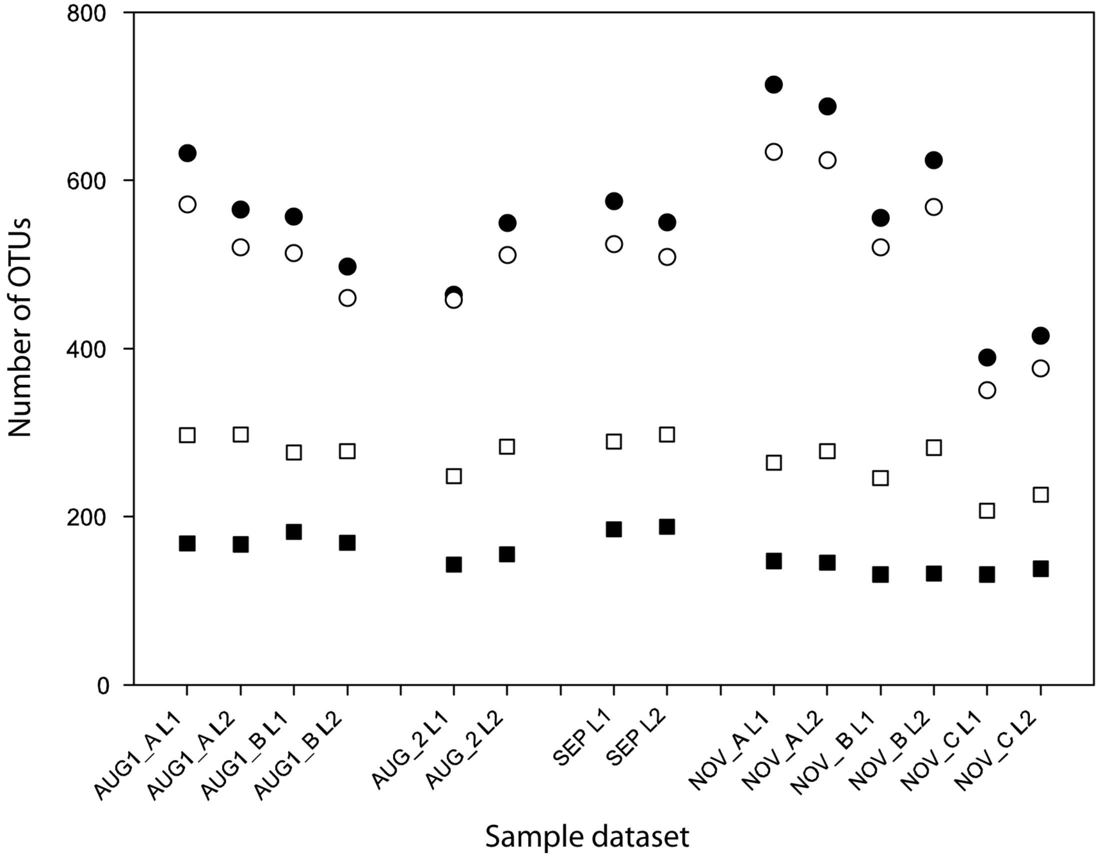

Figure S2. Number of OTUs identified in each dataset. OTUs identified by binning 16S rRNA amplicon sequences at the 97% (white) and 99% (black) levels after subsampling each dataset to the same depth (circles) and after filtering out singletons (squares). L1 and L2 in the name of each dataset indicate the independent sequencing replicates of the same DNA pool.

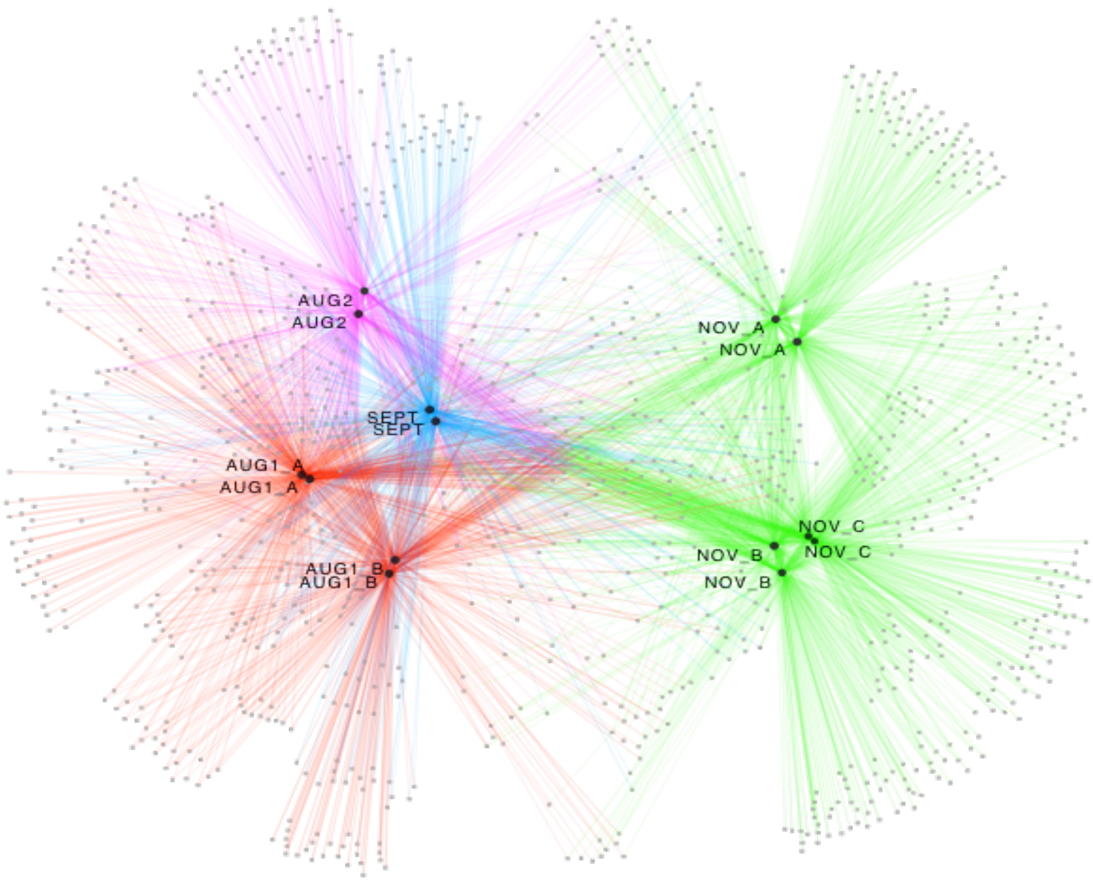

Figure S3: Similarity of datasets based on shared OTUs. The network shows the datasets clustered based on shared OTUs and the number of reads comprising each OTU. Datasets are indicated by a black circle and the connections are color coded by timepoint (Red= AUG1, Pink= AUG2, Blue= SEPT,and Green= NOV). OTUs are represented by grey squares. Duplicate sample runs were significantly connected to each other (G-test, p<0.01).

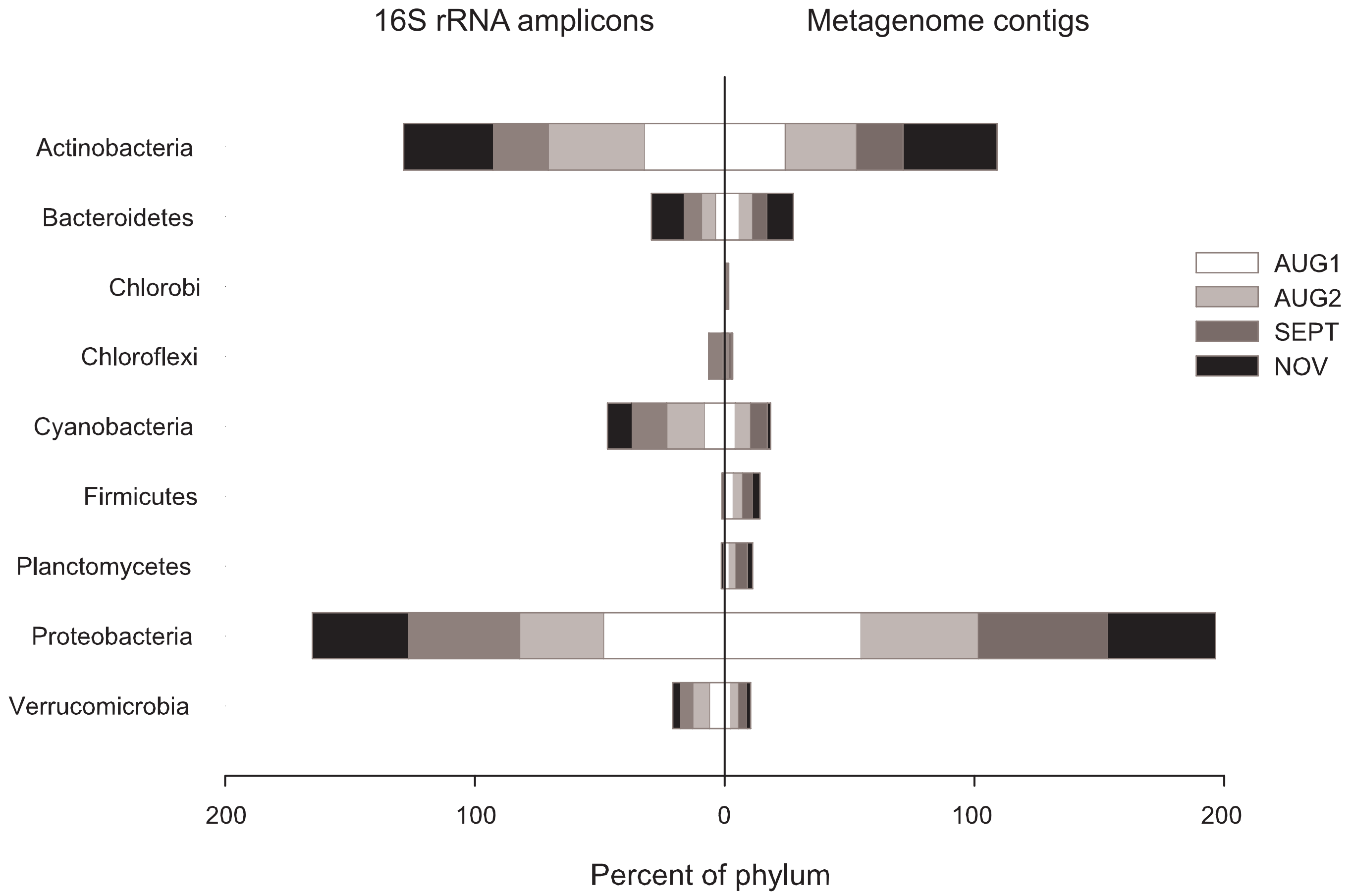

Figure S4. Differences in phylum abundance based on 16S and metagenomes. Phylum-level taxonomic affiliation (based on NCBI taxonomy) of the 16S rRNA gene amplicon sequences datasets (left) and metagenomic contigs (right) from the four timepoints (AUG1, white; AUG2, light grey; SEPT, dark grey; NOV, black).

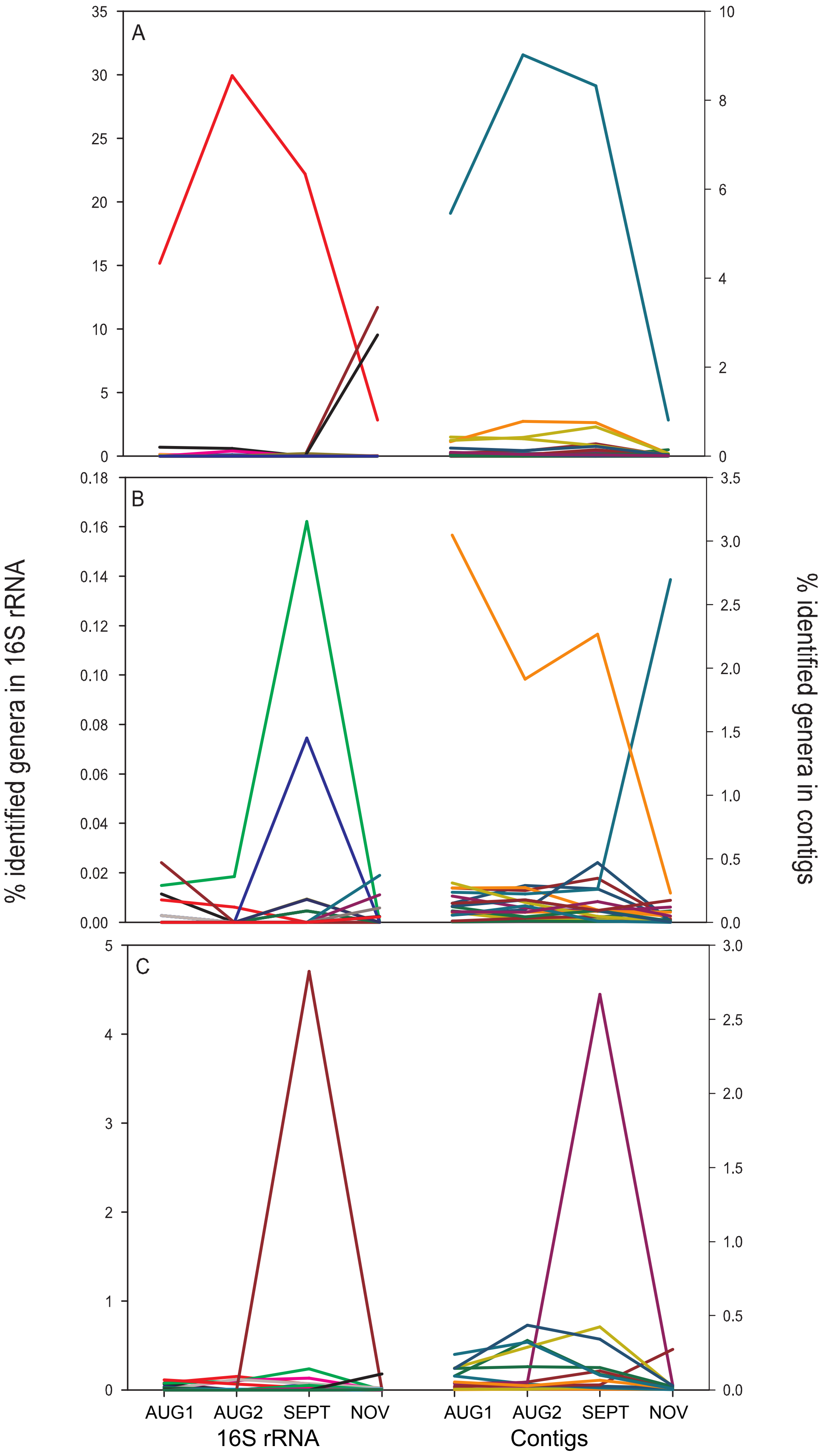
A
B
C
% identified genera in 16S rRNA
% identified genera in contigs
AUG1
AUG2
SEPT
NOV
16S rRNA
Contigs

Figure S5. Differences in abundance of individual genera based on 16S and metagenomes. The graph shows the abundance of all individual OTUs (16S data; left) or genomic populations (metagenomics data; right) attributed to *Synechococcus* (A), *Burkholderia* (B), and *Legionella* (C) genera.

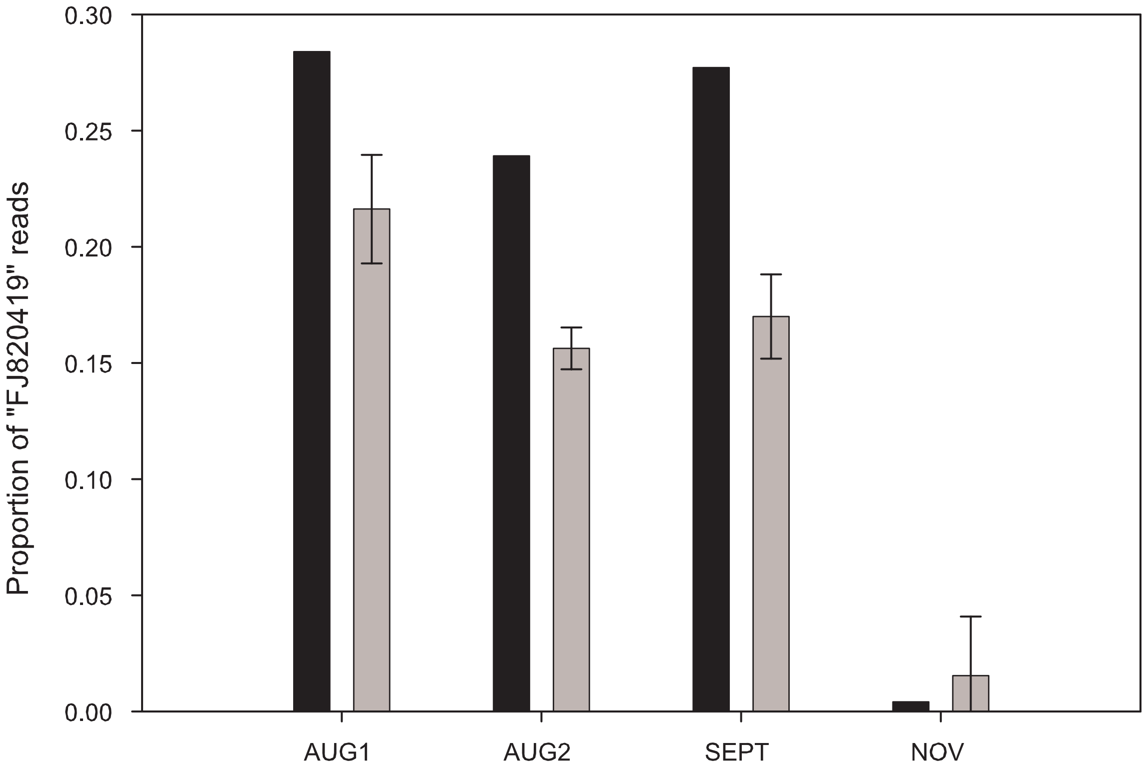

Figure S6. Abundance of an individual *Burkholderia*-like population based on 16S amplicon vs. 16S metagenomic reads. Bars represent the proportion of 16S rRNA gene reads in either the metagenomic datasets (black) or the 16S rDNA amplicon datasets (gray) that matched a full-length 16S rRNA gene sequence ("FJ820419") reconstructed from the metagenomic data using EMIRGE (2). The 16S amplicon error bars represent one standard deviation from the mean based on the replicate amplicon datasets.

References

1. **Oh S, Caro-Quintero A, Tsementzi D, Deleon-Rodriguez N, Luo C, Poretsky R, Konstantinidis KT.** 2011. Metagenomic insights into the evolution, function, and complexity of the planktonic microbial community of lake lanier, a temperate freshwater ecosystem. Appl. Environ. Microbiol. **77:**6000-6011.
2. **Miller CS, Baker BJ, Thomas BC, Singer SW, Banfield JF.** 2011. EMIRGE: reconstruction of full-length ribosomal genes from microbial community short read sequencing data. Genome Biol **12:**R44.
